# The Dental and Oral Science Magazine

**Published:** 1878-04

**Authors:** 


*The Dental and Oral Science Magazine.*—Quarterly; Devoted to Professional Interests and the Transactions of the New York Odontological Society.

This is the title of a New Dental Journal, which professes to be the only medium of publication for the proceedings of the New York Odontological Society, besides original articles from other sources.

It is stated that each number will contain about seventy-two pages in the literary department, and a number of advertising pages extra.

The first number has, besides the advertisements, about ninety pages, illustrated with seventeen extra fine wood cuts. The new publication presents a very handsome appearance, and will no doubt be ably managed.

